# Unveiling the Thermotolerance and Growth-Promoting Attributes of Endophytic Bacteria Derived from *Oryza sativa*: Implications for Sustainable Agriculture

**DOI:** 10.3390/microorganisms13040766

**Published:** 2025-03-27

**Authors:** Wonder Nathi Dlamini, Wei-An Lai, Wen-Ching Chen, Fo-Ting Shen

**Affiliations:** 1 Department of Soil and Environmental Science, National Chung-Hsing University, Taichung 402, Taiwan; boweph@gmail.com; 2 International Ph.D. Program in Environmental Sciences and Technology, University System of Taiwan, Taipei 11221, Taiwan; 3 Institute of Environmental and Occupational Health Sciences, National Yang Ming Chiao Tung University, Taipei 11221, Taiwan; 4 International Bachelor Program of Agribusiness, National Chung-Hsing University, Taichung 402, Taiwan; julychen@dragon.nchu.edu.tw; 5 Innovation and Development Center of Sustainable Agriculture (IDCSA), National Chung Hsing University, Taichung 402, Taiwan

**Keywords:** microbial endophytes, rice adaptation, heat stress, bioinoculants, crop resilience, microbial farming, regulatory phytohormones

## Abstract

High temperatures pose significant challenges to rice plants’ growth and their associated endophytic bacteria. Understanding how these bacteria respond to heat stress is vital. We assessed the potential of five endophytic bacterial strains derived from *Oryza sativa*—*Bacillus tequilensis* LB3, *B. coagulans* LB6, *B. paralicheniformis* AS9, *B. pumilus* LB16, and *B. paranthracis* i40C—to mitigate heat stress effects on rice plants. These strains demonstrated robust abilities in producing indole-3-acetic acid (IAA) and siderophores, nitrogen fixation, and solubilization of phosphate and potassium. Under high-temperature conditions, they significantly enhanced rice plant growth, with increases in plant length of up to 78% at 40 °C. Notably, LB6 showed the highest biomass increase (195%). The strains also improved chlorophyll SPAD values, an indicator of reduced heat stress effects and improved plant health. Phytohormone profiling and biochemical analyses revealed significant increases in abscisic acid (ABA) levels, reduced lipid peroxidation (MDA), and elevated osmoprotectant proline accumulation under heat stress. Inoculated plants exhibited up to 539 ng g^−1^ of ABA (vs. 62 ng g^−1^ in uninoculated controls), a 68% reduction in MDA (indicating less oxidative damage), and enhanced proline synthesis, collectively suggesting improved stress adaptation. These changes were linked to bacterial IAA production and nutrient modulation, which alleviated heat-induced physiological decline. These findings underscore the potential of these endophytes as biofertilizers to improve rice resilience under heat stress. Among the strains, LB6 exhibited superior performance, offering the greatest promise for heat-stress mitigation in rice production. This study advances our understanding of phytohormonal, heat stress signaling, and chemical processes underlying bacterial-mediated thermotolerance, providing a foundation for sustainable agricultural strategies. Future research can explore morphological and biochemical analyses, stress-responsive gene expression (e.g., HSPs, DREBs, and APX) linked to thermotolerance, and the combined effects of selected strains with fertilizers in high-temperature rice cultivation.

## 1. Introduction

Rice (*Oryza sativa*) is a fundamental food source for more than half of the global population, constituting 35–75% of daily calorie intake for over 3 billion people [1]. However, rice production struggles to meet the increasing demand in numerous countries, particularly in Africa, where a substantial portion of rice is imported. These limitations encompass challenges like crop protection, expensive inputs, insufficient knowledge, heat stress, and water scarcity. One of the most pressing ecological concerns impacting rice cultivation worldwide is temperature, with climate change leading to rising global temperatures. The year 2020 witnessed temperatures exceeding pre-industrial levels, underscoring the urgency of climate change [2]. Elevated temperatures can result in a significant decrease in agricultural production, particularly in regions like Asia, Africa, and the Middle East [3]. The optimal temperature range for rice cultivation is approximately 25 °C, with temperatures above 35 °C negatively affecting rice growth [4].

Plant phenology is profoundly influenced by weather conditions, as organisms adapt their life cycles in response to climate change [5]. Microbial endophytes, capable of colonizing living rice tissues, play a pivotal role in enhancing plant growth, enriching yields, and mitigating pathogen attacks [6]. These endophytic bacteria, accessible through surface-disinfected plant materials, have the potential to influence various aspects of rice development [5]. Endophytes colonize inner plant layers, and once they reach the xylem, they can circulate throughout the entire plant system [7]. The positive interactions between microbial endophytes and rice plants result in improved growth, pathogen suppression, and nutrient mobilization. These endophytes exhibit diverse competencies, specializations, and adaptive mechanisms [8]. Researchers have employed plant growth-promoting endophytic bacteria (PGPEB) to enhance the growth of various plants, including sorghum, chickpea, wheat, tomato, and potato [9]. These endophytes can confer resistance to a wide range of biotic and abiotic stresses, including heat. Although plant responses to high temperatures have been well documented, the reactions of endophytic bacteria to heat remain unclear [10,11].

The increasing global temperatures necessitate a deeper understanding of the intricate relationship between temperature stress, endophytic bacteria, and their combined impact on rice plant growth. Hence, this study seeks to understand how endophytic bacteria react to high temperatures and how these reactions influence the host plant. Additionally, we aim to investigate the physical and chemical changes within the plant caused by exposure to high temperatures, possibly attributed to endophytic bacteria. Ultimately, our goal is to mitigate the adverse effects of high temperatures on plant growth by harnessing the potential of endophytic bacteria. In essence, this research contributes to addressing the crucial issue of rice production sustainability in the face of climate change and growing global food demands.

## 2. Materials and Methods

### 2.1. Isolation and Screening of Thermotolerant Endophyte Bacteria Isolates

The rice seeds (*Oryza sativa* L. cv. TAINAN NO. 11) used in this study were collected from the Agricultural Experiment Station (AES) at National Chung Hsing University (NCHU), Chiayi Agricultural Experiment Station under the Taiwan Agriculture Research Institute, Council of Agriculture (COA), and two different agricultural estates in Taichung County, Taiwan. All isolated endophytes came from the seeds of rice from the 4 different sites. Rice seeds used for the Petri dish and soil pot experiment were from the Taichung agricultural estate.

To isolate thermotolerant endophytic bacteria, a total of 7.4 g of rice cv. TAINAN NO. 11 seeds were collected from four different sites. Approximately 1.9 g of seeds from each site were surface disinfected by soaking in 400 mL of 1.3% sodium hypochlorite solution for 30 min and washed three times with sterile water. Surface-disinfected seeds were added to 2 mL sterile water and crushed by a macerator machine [11]. The solution was successively diluted 10-fold over a range between 10^−1^ and 10^−8^, and a 100 μL cell suspension was plated onto nutrient agar (NA; HiMedia) plates. Nutrient agar supports the growth of a broad range of endophytic bacteria since it contains numerous nutrients essential for the development of microorganisms [12,13]. Plates were incubated at 40 °C and 45 °C for 5 d for the selection of thermotolerant bacteria. Isolates were stored in 2 duplications, 1 at −80 °C and 2 at −20 °C. The bacterial strains were named following a specific convention based on their locations. The beginning letters represent the collection site: DF and LB correspond to two different agricultural estates in Taichung County, AS denotes strains from the Agricultural Experiment Station at National Chung Hsing University, and isolates from the Chiayi Agricultural Experiment Station include the letter C at the end of the strain name.

### 2.2. Germination Bioassay for Evaluating Bacterial Inoculation Effects on Heat-Stressed Rice Seeds

A seed germination bioassay was conducted. A total of 127 thermotolerant endophytic isolates were tested for conferring rice with thermotolerance and growth promotion via a seed germination bioassay. Seeds were soaked in 200 mL of 1.25% (*w*/*v*) sodium hypochlorite solution for 30 min and washed three times with sterile water. While conducting this step, floating seeds were removed and thrown away. Later, seeds were soaked in sterile water for 24 h. Surface-sterilized seeds were immersed in 6 mL cell suspensions with constant cell density (A_600_ = 0.1) in a Petri dish containing 70 mm sterile filter paper. Each Petri dish contained 10 seeds which were replicated 5 times, making a total of 50 seedlings and an additional two kinds of controls. One control was for growth at 25 °C without heat shock, and another two controls included seeds growing in both heat shock conditions at 40 and 45 °C without bacterial inoculation. Sterile clicks were used to put 10 seeds on each of the 5 Petri dish plates for each of the 127 strains. The corresponding strains isolated under 40 °C (66 strains) and 45 °C (61 strains) were used to inoculate seeds for a 5 h heat shock at 40 °C and 45 °C, respectively, after growing at 25 °C for 5 d and then 5 d at 25 °C. Seeds were also tested under normal growth without treatment. Shoot and root lengths and shoot and root fresh weights were measured and recorded.

### 2.3. 16S rRNA Gene Sequence Analyses

Isolates that showed better growth at 45 °C or 40 °C heat shock were subjected to 16S rDNA sequence analysis. The following 18 strains were identified out of 77 strains isolated from two Dali district farms: DF3, DF9, DF11, DF18, DF19, DF31, DF36H, DF47, DF48, LB3, LB6, LB10, LB11, LB15, LB16, LB19, LB21, and LB24; 6 strains were identified out of 28 strains isolated from Agricultural Experiment Station in National Chung Hsing University (NCHU): AS5, AS9, AS10, AS16, AS23 and AS25; 4 strains were identified out of 22 strains from Chiayi Agricultural Experiment: a45C, f45C, g40C and i40C. Each isolate was grown and identified according to Williams and other researchers [14]. Genomic DNA was extracted using the UltraClean Microbial DNA Isolation Kit. The 16S rRNA genes were amplified via polymerase chain reaction with 27F: 5′-AGA GTT TGA TCC TGG CTC AG-3′ as the forward primer and 1492R: 5′-GTT TAC CTTGTT ACG ACT T-3′ as the reverse primer [15]. The conditions for polymerase chain reactions were as follows: 95 °C for 5 min, through 29 cycles of 95 °C for 1 min, 60 °C for 110 sec, 72 °C for 2 min, and then 72 °C for 7 min. A BLAST search (NCBI BLAST+ v2.11.0+, https://blast.ncbi.nlm.nih.gov/Blast.cgi accessed 15 July 2021) was used to compare the 16S rRNA gene sequences against the NCBI database. Closely related type strains were retrieved from GenBank using the EzBioCloud server (https://www.ezbiocloud.net/, accessed on 20 July 2021). Phylogenetic analysis was performed using the neighbor-joining (NJ) method with the appropriate DNA substitution model, and the statistical confidence of the nodes was assessed by bootstrapping with 2000 replications in MEGA X (version 10.2.6) [16]. The 16S rDNA sequences were deposited in GenBank under accession numbers MZ144276–MZ144281 and MZ340274–MZ340638. About 8 strains were selected for the PGP traits test after 16S rRNA gene sequence-based identification of 28 cultured TAINAN NO. 11 rice seed endophytic bacterial strains. The selected strains were different from each other and previously displayed better results in a seed germination bioassay under heat shock.

### 2.4. Evaluating Thermotolerant Endophytic Bacteria for Plant Growth-Promoting Traits

The strains LB6, AS9, i40C, AS10, LB3, LB16, DF36, and DF18 were chosen to test PGP traits comprised phosphate and potassium solubilization, siderophore, and IAA production and nitrogen fixation.

An acetylene reduction assay was used to test the nitrogen fixation ability of bacteria [17]. Isolates were streaked on nutrient agar (Himedia) plates and cultivated for 3 d. Later, the isolates were each inoculated into 10 mL semi-solid nitrogen-free medium in 20 mL test tubes. Incubation of the isolates was conducted at 30 °C for 4 d. Then, the covered lids of tubes were replaced by serum corks. Then, 2 mL standard acetylene gas was injected using a syringe to replace the air inside the test tubes. Later, the solution was kept for 1 day at room temperature. Then, 0.5 mL of gas was sucked from the tube and injected into the gas chromatography (Model 163; HITACHI) device to quantify the ethylene. The conditions of the analysis were as follows: carrier gas: nitrogen; flow rate: 35 mL h^−1^; temperature of the flame ionization detector (H2FID): 110 °C; column (1 m × 2 mm steel column packed with Porapak-T 80-100 mesh) temperature: 80 °C.

Thermotolerant endophyte bacterial isolates were detected for phosphate solubilization activities by growing them on Pikovskaya medium with tricalcium phosphate as the sole phosphate source [18]. Bacterial strains were placed in the central part of the 4 parts of each medium plate using sterilized tips. Different plates were incubated at 30 °C for 7 d, 40 °C for 7 d, and 45 °C for 7 d to monitor the positive or negative clear halo around the colonies.

Thermotolerant endophytic bacterial isolates were tested for potassium solubilization properties on Aleksandrow medium with potassium alumino silicate as the sole potassium source [19,20]. Plates were incubated at 30 °C for 7 d, 40 °C for 7 d, and 45 °C for 7 d to monitor the positive or negative clear halo around the colonies.

Thermotolerant endophyte bacterial isolates were tested for IAA-like compound production ability. Each bacterial strain was inoculated into test tubes containing 4 mL NB (HiMedia) containing 0.25 mg mL^−1^ tryptophan and incubated at 30 °C on a 150 rpm orbital shaker for 3 d. Then, an additional 1-day incubation was conducted at different temperatures (30, 40, and 45 °C, respectively). To test IAA production, centrifugation was run at 4000× *g* at 28 °C for 30 min, and 2 mL of supernatant was mixed with 4 mL of Salkowski reagent and placed in the dark for 20–30 min at room temperature. The absorbance of the solution was measured at 530 nm, and IAA concentrations were calculated by calibration of the standard IAA [21].

Thermotolerant endophytic bacterial isolates were detected for siderophores production ability on CAS blue agar as described by Schwyn and Neilands (1987); in doing so, two solutions were prepared and named solutions A and B which were later mixed [22,23]. Different plates were incubated at 30 °C for 7 d, 40 °C for 7 d, and 45 °C for 7 d to observe the affirmative or negative growth of a clear corona near the colonies.

### 2.5. Soil Pot Experiment

After multiple PGP trait tests among 8 endophytic bacterial isolates, 5 isolates showed good PGP traits and were selected for evaluation in the soil pot trial with rice plants. The soil pots used had a base with a 6 cm diameter, a top with an 8 cm diameter, and the height was 15 cm. Each was filled with 600 g of air-dried, sieved (2 mm mesh), and homogenized soil. The soil properties were determined as follows: soil texture was evaluated using the hydrometer method; soil pH and electrical conductivity were detected by suspending the soil in deionized water (1:5, *w*/*v*); total soil N was measured using Kjeldahl’s digestion method [24]; other soil nutrients (P, K, Ca, Mg, Fe, Mn, Cu, and Zn) were extracted using Mehlich no. 3 solution and analyzed through inductively coupled plasma-atomic emission spectrometry by using a sequential Jobin Yvon JY 138 Ultrace spectrometer; and soil organic matter was weighed using the loss-on-ignition method [25]. The soil physicochemical properties were as follows: texture: sandy loam (68.6% sand, 12.0% clay, and 19.4% silt); pH: 7.45; electrical conductivity: 284.25 µS cm^−1^; organic matter: 2.67%; total nitrogen: 0.04%; 171, 196, 2641, 320, 535, 25, and 0.95 mg kg^−1^ for Melich’s P, K, Ca, Mn, Fe, Cu, and Zn, respectively.

Seeds were surface disinfected and washed. Floating seeds were removed and thrown away. Approximately 20 surface-sterilized seeds were soaked in a Petri plate comprised 10 mL cell suspensions (A_600_ = 0.8) from 4-day old colonies on 70 mm sterile filter paper and incubated at 25 °C for 4 d in the dark. Each treatment was replicated 5 times. Other surface-sterilized seeds soaked in 10 mL sterile water were used as the control. Five strains, LB3, LB6, AS9, LB16, and i140C, were separately inoculated and a no inoculation sample was used as a control. There were 10 seedlings planted in each pot with five replicates for each treatment. For each pot, 10 sprouts inoculated with one strain were planted and replicated 5 times, resulting in 50 seedlings for each strain and an additional 50 uninoculated seedlings for the control. Two separate experiments were conducted for a duration of 21 d at two different temperatures, 25 °C and 40 °C, in a growth chamber (GC-559) with a 12 h photoperiod (light: 153–179 µmole photons s^−1^ m^−1^). Rice seedlings were grown at 25 °C (30 pots) and 40 °C (30 pots) separately.

### 2.6. Measurement of Plant Growth Parameters

Shoot and root lengths, chlorophyll content in leaves (SPAD), shoot and root dry weights, and physicochemical processes within the plants, which are crucial for the wellbeing of the plants and offer necessary dynamism for survival, especially under heat stress conditions, were measured after 21 d.

In a Petri dish test, shoot and root lengths and the fresh weights of shoots and roots were measured in a total of 6750 seedlings and this included 5 replicates of each of the 127 isolates (50 per isolate since there were 10 in each of the 5 pots) and two control experiments (test without inoculation under heat shock or normal temperature). Roots and shoots were detached using a pair of scissors under a Petri dish and the lengths of the roots and shoots were measured instantaneously, and the fresh weights of the roots and shoots were measured. In the soil pot experiment, plant length and fresh weight were measured for a total of 300 plants. The dry weights of the shoots and roots were recorded after drying in an oven at 70 °C for 3 d. The leaves were counted. Counting several roots and measuring their lengths involved cutting off the plastic pot and washing off the soil.

### 2.7. Analyzing Physiological Parameters in Response to Stress

Chlorophyll content was measured with a chlorophyll meter (SPAD 502Plus, KONICA MINOLTA) which is a simple, rapid, and accurate technique for measuring chlorophyll in SPAD values. Compared to other ways of determining SPAD values, such as using spectrophotometric measurements of leaf samples, the chlorophyll meter is the best since the other ways are time-consuming, damaging, and costly, and for that reason, other methods such as in vitro determinations may not be appropriate for this experiment. Understanding the dynamics that have an impact on the SPAD interpretation is essential to improve its uses. These factors comprise the leaf location, environment of the leaf being studied, rice variety, and the growth phase. For each treatment, 20 leaves were measured from 5 soil pots.

The content of proline was determined by using an ethanolic extract (1% ninhydrin in 60% acetic acid + 20% ethanol) which was kept at 4 °C with 0.05 mm stainless-steel beads in a 2 mL tube to break fresh plant tissue after measuring the plant weight. A laboratory vortex machine was used to shake the mixture for 10 min, and later, 0.5 mL of supernatant was removed and mixed with 1 mL ethanolic extract in a 2 mL tube in a 2:1 proportion. The reaction combination was heated in a water bath at 95 °C for 20 min, and after cooling to room temperature, the absorbance was measured spectrophotometrically (U-3010) at 520 nm [26,27].

To measure the degree of lipid peroxidation, malondialdehyde (MDA) amounts in rice plants under normal temperature (25 °C) and heat stress (40 °C) were analyzed. Plant tissues were cut and placed inside a tube containing 1.5 mL of 80% ethanol with stainless steel beads, vortexed vigorously for 10 min, and centrifuged at 3000× *g* for 10 min. Two sets of tubes were used to put 0.5 mL of supernatant; one contained 1 mL no-TBA and the other contained 1 mL TBA. These two were vortexed, heated in a water bath at 95 °C for 25 min, and the absorbances at 440, 532, and 600 nm were measured spectrophotometrically. MDA levels (nmol·mL^−1^) were calculated according to the following formula: (A_532+TBA_ − A_600+TBA_) − (A_532−TBA_ − A_600−TBA_) = A, (A_440+TBA_ − A_600+TBA_)0.0571 = B, MDA equivalents (nmol·mL^−1^) = (A − B/157,000) 10^6^ [28], and hence MDA (µmol·g^−1^ leaves) was calculated [29].

For quantification of the abscisic acid (ABA) in rice plants, the extraction procedure was conducted according to the description in [30] with slight modification [31]. The rice leaves were cut, weighed, and put in the tube containing 0.5 mm diameter stainless-steel beads, and 1 mL of a solution containing 80% (*v*/*v*) methanol, 0.1% (*w*/*v*) butylated hydroxytoluene (antioxidant), and 0.02 M sulfuric acid was added and was vortexed vigorously for 10 min. The tubes were centrifuged at 10,000× *g* at 4 °C for 15 min, and later, the absorbance of the supernatant at 261 nm was measured spectrophotometrically. The ABA content was calculated according to the Beer–Lambert law since the molar absorption coefficient of ABA (in 96% ethanol) at 261 nm is 22,000 L^−1^ mol^−1^ cm^−1^ [32,33].

### 2.8. Statistical Analysis

The data of this experiment have been expressed as the means with the standard errors (Mean ± SD). Statistical analysis between the mean values of controls, inoculated heat-stressed, and unstressed rice plants was executed by using SPSS 25 (IBM) with one-way ANOVA and a post hoc test with the LSD test. *p* < 0.05 represents significance. A two-way analysis of variance (ANOVA) general linear model (univariate) was used to evaluate the main effects of the treatment factors (heat stress and bacterial inoculation) and the interactions among the treatment factors (F-values) on shoot and root lengths and dry weights, shoot proline, chlorophyll SPAD, proline, MDA, and abscisic acid contents.

## 3. Results

### 3.1. Isolation of Thermotolerant Bacteria and Growth Temperature Test

A total of 127 thermotolerant endophytic isolates were isolated from surface-sterilized rice (*Oryza sativa* L. cv. TAINAN NO.11) seeds and screened for their ability to grow at 40 °C (Table S2) and 45 °C (Table S1) on solid nutrient agar (NA) media for 5 days. Among these isolates, 66 strains exhibited growth at 40 °C, including 11 from C, 17 from AS, 25 from DF, and 13 from LB. Additionally, 61 isolates grew at 45 °C, with 11 from C, 11 from AS, 23 from DF, and 16 from LB. The results show that increasing the temperature from 40 °C to 45 °C hindered the growth of isolates.

### 3.2. Seed Germination Bioassay

All 127 isolates were used for the Petri dish trial, and some isolates significantly improved the lengths and fresh weights of the hypocotyl and radicle under heat shock temperatures at 40 °C and 45 °C, while other isolates caused a decrease in hypocotyl and radicle lengths and fresh weights (Tables S1 and S2). The hypocotyl of inoculated seeds showed a significant increase when compared with uninoculated seeds (BK) which suggests that nine endophytic bacterial isolates, namely DF36, LB15, LB16, LB19, LB21, LB24, AS23, AS25, and i40C, significantly increased hypocotyl lengths under 40 °C heat shock by 2-fold, while three other isolates, namely DF31, DF47, and DF48, increased hypocotyl lengths by 1-fold (Table S2). The seeds inoculated with DF31, DF36, LB11, LB15, LB16, LB19, LB24, AS23, AS25, and i40C showed an increase of 2-fold in radicle length while seeds inoculated with isolates DF47 and DF48 showed a significant increase of 1-fold in radicle lengths under 40 °C heat shock. According to the results, hypocotyl and radicle lengths under heat shock of 40 °C without inoculation (BK) were affected by 1.7-fold and 1.5-fold, correspondingly, when compared with normal growth (NG), which represented uninoculated seeds without heat shock. The seeds inoculated individually with isolates LB11, LB15, LB16, AS23, and AS25 showed a significant 3-fold increase in hypocotyl fresh weight while seeds inoculated with isolates DF31, DF36, DF47, LB19, LB24, AS25, and i40Cs displayed a significant 2-fold increase in hypocotyl fresh weight, and isolate DF48 improved hypocotyl fresh weight by 1-fold under 40 °C. Under the same condition of heat shock at 40 °C, seeds inoculated with strains LB11, LB15, LB16, and LB19 individually improved by 3-fold the radicle fresh weight, while the seeds inoculated with isolates DF31, DF36, DF47, LB24, AS23, and AS25 exhibited significantly increased, by 2-fold, radicle fresh weight, and isolates DF48 and i40C significantly improved radicle fresh weight by 1-fold.

Inoculation with bacteria resulted in a significant increase in hypocotyl and radicle lengths for inoculated seeds under 45 °C heat shock (Table S1). Inoculation with isolate a45C resulted in a 3-fold increase in both radicle and hypocotyl lengths, whereas 10 isolates, namely DF9, DF18, DF19, AS5, AS9, AS10, LB3, LB6, LB10, and F45C, improved radicle and hypocotyl lengths 2-fold, while isolate DF11 increased radicle and hypocotyl lengths by 1-fold. According to the results presented here, we knew that there was a significant increase in fresh weights for both the hypocotyl and radicle in inoculated seeds. The results suggest that hypocotyl and radicle fresh weights under heat shock of 45 °C in the control (BK) were affected 2-fold and 3.6-fold, respectively, when compared to normal growth (NG). Hypocotyl and radicle lengths from uninoculated seeds under 45 °C heat shock were affected by 2.1-fold and 1.8-fold, respectively. The results suggest that the effects of 45 °C heat shock are greater than the 40 °C effects, and this can be seen through the hypocotyl and radicle fresh weights and lengths. Using endophytes offers the greatest yield in rice plants and decreases ecological influences on rice plants. One major advantage of endophytes is that they live inside the rice plant for most of their lifetime not exerting any harmful influence on the rice plant.

### 3.3. Screening and Identification of Thermotolerant Endophytic Bacteria

**Figure 1:**
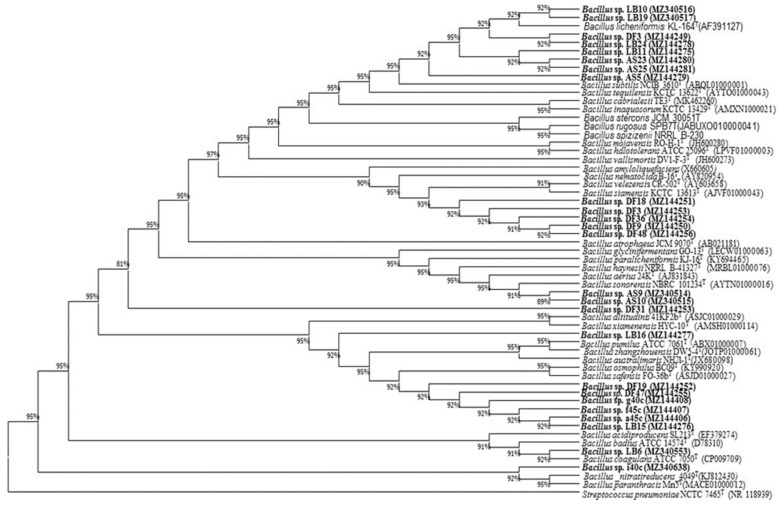
Phylogenetic tree of 25 endophytic bacteria endophytes (numbers at forks are confidence fractions after 2000 repeats in the bootstrap analysis).

There were 25 strains chosen for identification out of the 127 strains isolated from rice seeds based on their results in the Petri dish trial. The results were obtained after 16S rRNA genes were amplified, sequenced, and matched using a record of identified 16S rRNA gene sequences and BLAST. The 16S rDNA sequences of 25 endophytic bacterial strains displayed more than 97% matching with the closest BLAST sequence, hence supporting the significant hypothesis which states that sequences of more than 97% distinctiveness denote similar species (Table S3 and Figure 1). The results revealed the following isolates per species based on the 16S rRNA gene sequence: AS25, AS23, AS5, LB24, LB19, LB10, LB11, and LB3 with *Bacillus tequilensis* (99.43%≤), AS10 with *B. haynesii* (99.71%), AS9 with *B. paralicheniformis* (99.64%), LB16 with *B. pumilus* (99.72%), LB15, DF47, DF19, G40c, f45C, and a45C with *B. safensis* (99.86%≤), LB6 with *B. coagulans*(99.73%), DF48, DF36, DF31, DF18, DF9, and DF3 with *B. velezensis* (99.71%≤), and i40C with *B.paranthracis* (99.93%). The 16S rDNA sequences obtained in the present study were deposited in the GenBank database under Accession Numbers MZ144276, MZ144406, MZ144407, MZ144408, MZ144255, MZ144252, MZ144277, MZ144253, MZ340553, MZ340638, MZ340514, MZ340515, MZ144251, MZ144249, MZ144254, MZ144250, MZ144256, MZ340516, MZ340517, MZ340279, MZ340274, MZ340278, MZ144275, MZ144280, and MZ144281.

### 3.4. Evaluating Nitrogen Fixation, Phosphate and Potassium Solubilization, IAA Production, and Siderophore Production

A total of eight endophytic bacterial strains belonging to different species (Table S3 and Figure 1) were selected for plant growth-promoting trait analysis based on their superior performance in preliminary heat stress tolerance assays (Tables S1 and S2). These isolates exhibited significantly enhanced seed germination rates, hypocotyl and radicle elongation, and fresh biomass accumulation under 40–45 °C heat stress compared to uninoculated controls. Their thermotolerance and growth-promoting potential under stress prompted further evaluation of their nitrogen fixation, phosphate and potassium solubilization, IAA production, and siderophore production capabilities (Table 1).

All eight isolates displayed multiple plant growth-promoting traits (Table 1). All endophytic bacterial strains presented positive results for nitrogen-fixing activity in the range between 0.01 nmole C_2_H_4_ h^−1^ for the weakest nitrogen-fixing activity (isolate i40C) and 0.38 nmole C_2_H_4_ h^−1^ for the strongest nitrogen-fixing activity (isolate AS10). About five out of the eight strains tested positive for phosphate solubilization ability. Isolates DF18 and DF36 showed negative results under 30 °C, positive results with a smaller clear zone under 40 °C, and positive results with a larger clear zone under 45 °C. Isolate LB6 displayed positive results with a small clear zone through the three sets of different temperatures. Isolate LB16 results displayed positive results for phosphate solubilization ability with a large clear zone under normal temperature (30 °C), a smaller clear zone under 40 °C, and negative results under 45 °C. Approximately three isolates showed positive results for potassium solubilization, while four isolates showed negative results.

All endophytic bacterial strains presented positive results for IAA production in the range between 1.74 mg mL^−1^ for the lowest production (isolate DF36) and 54.04 mg mL^−1^ for the highest production (isolate AS19). In some isolates (i.e., i40C), increasing the temperature increased production, while in other isolates it was the opposite (i.e., LB3), and there was no pattern observed with temperature increases or decreases in other isolates. All endophytic bacterial strains presented positive results for siderophore production. This study shows that eight endophytic bacterial isolates, namely LB6, AS9, i40C, AS10, LB3, LB16, DF36, and DF18, used to have multiple PGP traits which include the ability of nitrogen fixation, phosphorous and potassium solubilization, IAA, and siderophore production (Table 1). Nutrients improve the growth of plants under high-temperature conditions and help plants stand heat stress conditions. IAA can facilitate heat stress tolerance in plants by stimulating gene expression, improving regulation of photosynthetic pigment build up and osmoprotectant and antioxidant enzyme production. Siderophores in plants can be seen as biocontrols and have an essential part in improving plant growth. The multiple PGP traits (Table 1) likely explain how the isolates enhanced rice plant growth under heat stress, as observed in the seed germination bioassay (Tables S1 and S2).

### 3.5. Plant Growth States

**Figure 2:**
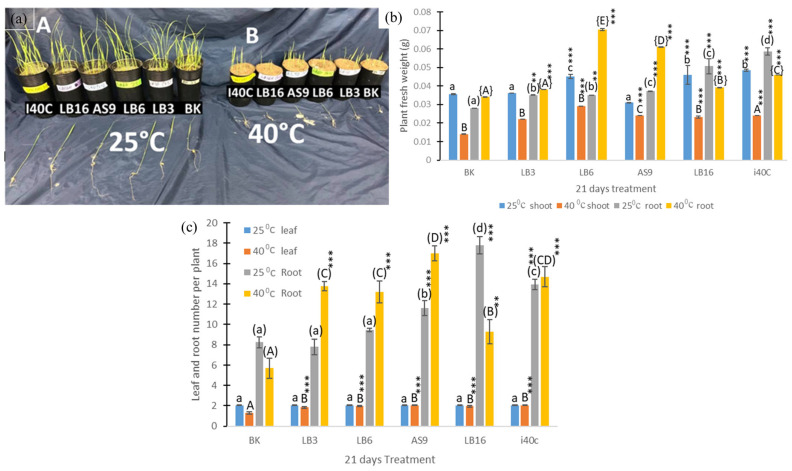
(**a**) Effect of 5 endophytic bacterial isolates on rice plant growth determined after 21 days ((**A**)—25 °C; (**B**)—40 °C); (**b**) plant fresh weight and (**c**) leaf and root numbers of rice plants under heat stress at 40 °C compared with normal growth at 25 °C after 21 days. Each data point is the mean of 5 replicates. Error bars represent standard errors. The bars represented with different letters are significantly different from each other. Statistical comparison was conducted within the same growth day, and statistically significant effects are indicated as ** *p* < 0.01, and *** *p* < 0.001.

**Figure 3:**
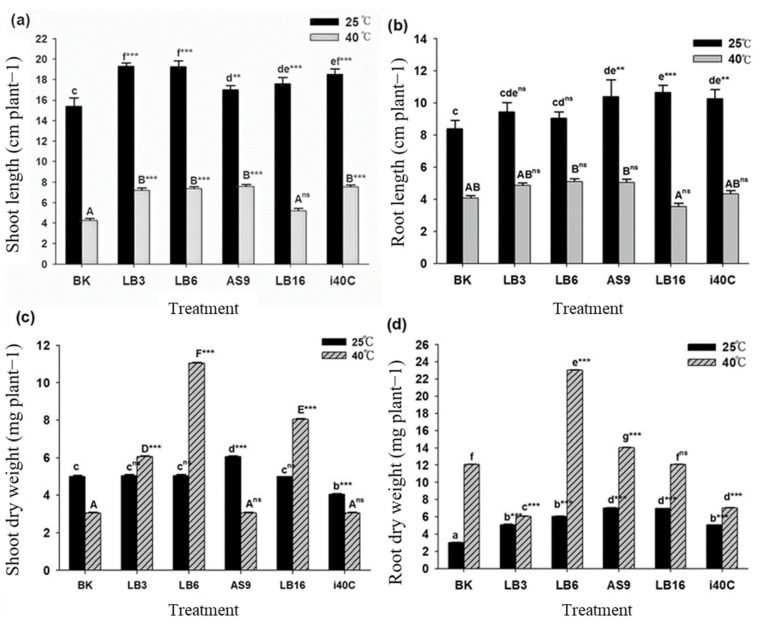
Effect of combination of heat stress (40 °C) and inoculation (strain LB3, LB6, AS9, LB16, or i40C) on (**a**) shoot and (**b**) root lengths and (**c**) shoot and (**d**) root dry weights of rice plants after cultivation for 21 days. Each data point is the mean of 5 replicates. Error bars represent standard errors. The bars represented with different letters are significantly different from each other. Statistical comparison was conducted, and statistically significant effects are indicated as a superscript of ns *p* ≥ 0.05, ** *p* < 0.01, and *** *p* < 0.001.

In comparison with the control (BK), isolate inoculation greatly improved the thermotolerance of rice plants as shown through the variations in lengths and the fresh and dry weights of shoots and roots (Figure 2 and Figure 3). The data showed that inoculation with endophytic bacteria isolates LB3, LB6, AS9, LB16, and i40C significantly increased the length of shoots by 25%, 25%, 10%, 14%, and 20%, respectively, (Figure 3a) and increased the length of the roots by 7%, 23%, 27%, and 22%, respectively, under normal temperature (Figure 3b).

Heat stress affected shoot length, and this can be seen from BK with a change after 21 d from 15.39 cm (25 °C) to 4.25 cm (40 °C); however, inoculation with endophytic bacteria isolates LB3, LB6, AS9, LB16, and i40C significantly increased the lengths of the shoots by 69%, 73%, 77%, 22%, and 76% (Figure 3a); while isolate LB16 decreased root length by 12%, isolates LB3, LB6, AS9, and i40C significantly increased shoot length by 19%, 25%, 25%, and 6%, respectively, under heat stress condition (Figure 3b). Overall, a significant increase in the lengths of inoculated plants was seen for shoots and roots under normal temperature (25 °C) with a 25% (LB3 and LB6) increase being the highest for shoots lengths and a 27% (LB16) increase being the highest increase for root length after 21 d.

After 21 d under heat stress, an increase in shoot and root fresh weight was seen in all rice plants inoculated with isolates LB3, LB6, AS9, LB16, and i40C with changes of 57, 107, 71, 65, and 71% for shoots and 12, 107, 79, 15, and 35% for roots, respectively (Figure 3b). Under normal temperatures, most inoculated plants displayed an increase in shoot and root fresh weight, with a 36% (i40C) being the highest increase for shoots, with 109% (i40C) as the highest increase for roots, though isolate AS9 resulted in a 13% reduction in shoot fresh weight. There was an increase in shoot and root dry weights of most inoculated plants under heat stress with 195% (LB6) as the highest increase for shoots (Figure 3c) and 91% (LB6) as the highest increase for roots (Figure 3d). Conversely, isolates LB3 and i40C resulted in a decrease in root dry weights by 50% and 41%, correspondingly. In normal growth, AS9 inoculation caused the highest increase in dry weight by 21% in the shoot (Figure 3c) and 133% in the root (Figure 3d), on the other hand, isolate i40C resulted in a 19% decrease in shoot dry weight under 25 °C. Inoculated and uninoculated plants recorded equal leaf numbers under 25 °C, but there was a variation under 40 °C whereby inoculated plants showed an increase in leaf numbers with isolate LB16 showing the highest increase of 1.6-fold (Figure 2c). There was also an increase in root numbers of inoculated plants at 25 °C and 40 °C, with isolate LB16 showing the highest increase of 1.8-fold under 25 °C and with isolate AS9 showing the highest increase of 2.5-fold under 40 °C.

### 3.6. SPAD Chlorophyll Analysis

**Figure 4:**
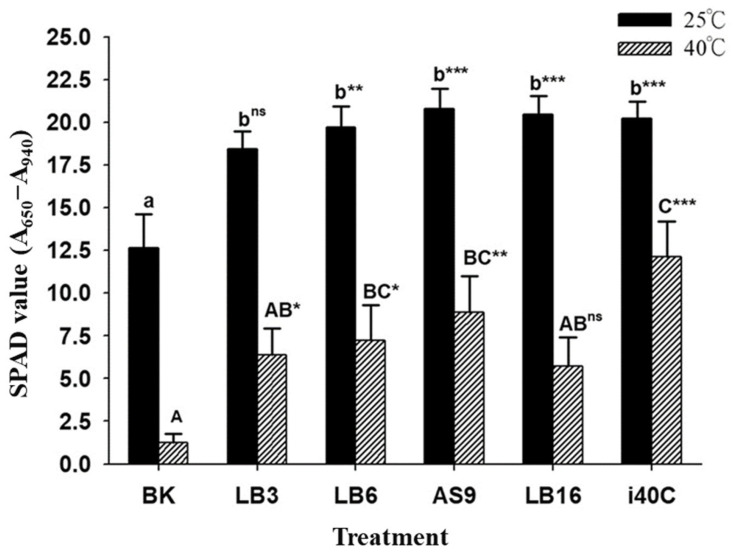
Effect of combination of heat stress (40 °C) and inoculation (strain LB3, LB6, AS9, LB16, or i40C) on chlorophyll contents in rice plants after cultivation for 21 days. Each data point is the mean of 5 replicates. Error bars represent standard errors. The bars represented with different letters are significantly different from each other. Statistical comparison was conducted within the same growth day. Statistical significance between each mean and BK is indicated as a superscript of ns *p* ≥ 0.05, * *p* < 0.05, ** *p* < 0.01, and *** *p* < 0.001.

The Soil Plant Analysis Development (SPAD) values under shoot chlorophyll outcomes revealed that heat stress significantly reduced the chlorophyll content; hence, after 21 days, the chlorophyll contents of uninoculated plants remained low (1.7) under heat stress at 40 °C, while chlorophyll contents immensely increased in all inoculated plants under the same heat stress condition (Figure 4). The results suggest that inoculation of thermotolerant endophytic bacteria isolates LB3, LB6, AS9, LB16, and i40C alleviated heat stress and improved chlorophyll SPAD values after 21 d from 1.3 to 6.4, 7.2, 8.9, 5.7, and 12.1, respectively. There was a great increase in chlorophyll contents in inoculated rice plants under the normal temperature of 25 °C, with the three strains AS9, LB16, and i40C showing the highest increase of approximately 153% compared to the control. Heat stress significantly lowered chlorophyll content in uninoculated plants, and isolates LB3, LB6, AS9, LB16, and i40C alleviated heat stress and improved chlorophyll content. Inoculation might have resulted in a change in nutrient distribution which improved rice plants’ growth.

### 3.7. Osmoprotectant Proline Content

**Figure 5:**
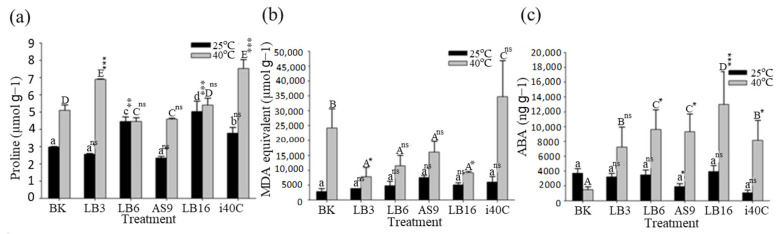
Effect of combination of heat stress (40 °C) and inoculation (strain LB3, LB6, AS9, LB16, or i40C) on (**a**) proline, (**b**) MDA, and (**c**) ABA contents after cultivation for 21 days. Error bars represent standard errors calculated from 4 replicates. The bars represented with different letters are significantly different (*p* < 0.05) from each other. Statistical significance between each mean from BK is indicated as ns *p* ≥ 0.05, * *p* < 0.05, ** *p* < 0.01, or *** *p* < 0.001.

Proline has been proposed to take part in plants’ osmoregulation stress tolerance. Under heat stress conditions, proline content was found to be significantly higher when compared to normal conditions. Except for plants inoculated with isolate LB6, the proline content was the same for both conditions, whereas all plant inoculation further increased proline content with isolate i40C resulting in the highest increase (Figure 5a). A less significant change in proline content in the shoot of rice plants growing under normal temperature was observed. The IAA production ability of isolate i40C has been seen to increase with increasing temperature (Table 1), and this suggests the reason for why proline content was the highest in plants inoculated with isolate i40C under heat stress conditions.

### 3.8. MDA Content

Total malondialdehyde (MDA) content in rice florae in heat stress as well as ordinary conditions was assessed to obtain the degree of lipid peroxidation (LPO). Under ordinary conditions (25 °C), the MDA content showed no significant difference between inoculated and uninoculated plants, whereas in heat stress conditions (40 °C), plants inoculated with strains LB3 and LB16 displayed significantly decreased MDA content by 68 and 63%, respectively, in comparison with uninoculated plants (Figure 5b). In the course of heat stress, MDA functions as a way to gauge the degree of lipid peroxidation in plants; hence, more MDA signifies more tissue mutilation, while less MDA signifies less tissue damage.

### 3.9. Endogenous Phytohormone Abscisic Acid (ABA)

The endogenous phytohormone ABA upholds viability in plants when they experience varying temperature conditions. This phytohormone enhances plants’ ability to adapt to heat stress and hence controls plant development. Under normal conditions (25 °C), an insignificant variation amount was seen in all inoculated and non-inoculated rice plants. In normal conditions, the ABA content was low. However, rice plants exposed to heat stress showed momentously increased ABA (Figure 5c). All rice plants inoculated with endophytic bacteria showed significantly higher ABA content than uninoculated plants under heat stress conditions. Under heat stress conditions, the average ABA content for uninoculated plants was 62 ng g^−1^, while for plants inoculated with isolates LB3, LB6, AS9, LB16, and i40C, average ABA content was 300, 398, 386, 539, and 337 ng g^−1^, respectively.

### 3.10. Effect of Heat Stress and Inoculation on Measured Variables

Table 2 summarizes the statistical significance of heat stress, bacterial inoculation, and their interaction on the measured variables. The results indicate that heat stress had a highly significant (*p* < 0.001) impact on all growth parameters, including shoot length, root length, shoot dry weight, and root dry weight. Bacterial inoculation also showed a significant influence, particularly on shoot dry weight and root dry weight. The interaction between heat stress and inoculation revealed varying levels of significance, suggesting that while inoculation mitigates stress effects on certain traits, its impact varies depending on the measured variable.

## 4. Discussion

Heat stress profoundly disrupted rice physiology, consistent with global reports of thermal stress impairing crop productivity through chlorophyll degradation and oxidative damage [34,35]. Bacterial inoculation counteracted these effects, particularly in shoot biomass and proline accumulation, likely via microbial production of osmoprotectants and phytohormones [36]. Proline emerged as a pivotal osmoprotectant under heat stress, stabilizing the cellular osmotic equilibrium and mitigating oxidative damage, a mechanism widely documented in plants facing abiotic stressors [37,38]. The elevated proline levels in inoculated plants align with studies demonstrating that *Bacillus* spp. enhance thermotolerance in crops like wheat and soybean by modulating osmolyte synthesis [9,39]. This synergy between bacterial inoculation and proline enrichment underscores its critical role in sustaining plant growth under thermal stress, offering a promising strategy to fortify crops against escalating global temperatures [40].

Concurrently, reduced malondialdehyde (MDA) content in inoculated plants under heat stress highlights the bacteria’s capacity to attenuate lipid peroxidation and oxidative tissue damage [41]. Similar findings have been reported in *Vitis vinifera*, where PGPR inoculation lowered MDA levels under freezing stress by enhancing antioxidant activity [42,43]. The efficacy of *Bacillus* strains in minimizing oxidative damage aligns with their known ability to stimulate antioxidant enzymes such as superoxide dismutase and catalase [42], further validating their role in redox homeostasis. Notably, the non-significant interaction between heat stress and inoculation for chlorophyll content suggests that bacterial strains may prioritize mitigating secondary stress symptoms (e.g., oxidative stress) over direct protection of photosynthetic machinery, a phenomenon observed in heat-stressed maize treated with *Pseudomonas fluorescens* [44,45].

Abscisic acid played a central role in mediating stress adaptation, with inoculated plants exhibiting elevated ABA levels under heat stress [46]. ABA’s dual function in stomatal regulation and the activation of antioxidant defense pathways is well established in drought and heat responses [47]. The synergy between bacterial inoculation and ABA upregulation mirrors findings by Ahmad et al. (2022), where PGPR-enhanced ABA signaling improved water-use efficiency and stress recovery in plants under drought stress [48]. This suggests that ABA serves as a key biochemical messenger in microbial-mediated stress resilience, bridging phytohormonal dynamics and cellular redox balance [49,50].

The interplay between heat stress and inoculation significantly influenced plant growth and stress markers, with five endophytic bacterial isolates, namely *Bacillus tequilensis* LB3, *B. coagulans* LB6, *B. licheniformis* AS9, *B. zhangzhouensis* LB16, and *Staphylococcus pasteurii* i40C, demonstrating exceptional efficacy in mitigating heat-induced damage. These isolates likely operate through multifaceted mechanisms, including nutrient solubilization, phytohormone production, and oxidative stress alleviation, consistent with the documented traits of PGPR in *Glycine max* and *Oryza sativa* [51,52]. Their ability to enhance stress tolerance while promoting growth positions them as viable candidates for biofertilizer development, particularly in regions vulnerable to climate-induced temperature fluctuations.

## 5. Conclusions

Elevated temperatures pose challenges to both rice plants and their associated endophytic bacteria, making it imperative to unravel the intricate morphological and biochemical responses induced by thermal stress in plants, as well as the reactions of endophytic bacteria to such conditions. Notably, the endophytic bacterial isolates investigated in this study, including *Bacillus tequilensis* LB3, *B. coagulans* LB6, B. paralicheniformis AS9, *B. pumilus* LB16, and *B. parantracis* i40C, have exhibited remarkable capabilities in mitigating the adverse effects of high-temperature stress on rice plants. These findings open up avenues for the development of biofertilizers leveraging these isolates, thereby enhancing the ability of rice plants to withstand heat stress and flourish. Furthermore, our results shed light on the intricate interplay between phytohormones, heat stress signaling pathways, and various chemical processes involved in the efficacy of plant growth-promoting endophytic bacteria. This knowledge holds promise for optimizing rice plant growth under heat-stress conditions, contributing to enhanced agricultural productivity in the face of escalating global temperatures.

## Figures and Tables

**Table 1:** Plant growth-promoting traits of eight endophytic bacterial isolates.

Strain	N Fixation (n Mole Ethylene h^−1^)	P Solubilization			K Solubilization			IAA Production (mg mL^−1^)			Production of Siderophore		
	30 °C	30 °C	40 °C	45 °C	30 °C	40 °C	45 °C	30 °C	40 °C	45 °C	30 °C	40 °C	45 °C
DF18	0.06 ± 0.02 ^a^	−	+	++	−	−	−	7.75 ± 0.1 ^b^	3.22 ± 2.71 ^a^	14.26 ± 0.32 ^e^	++	++	++
DF36	0.02 ± 0.01 ^a^	-	+	++	+	+	+	4.65 ± 0.29 ^a^	3.35 ± 0.06 ^a^	1.74 ± 0.33 ^a^	++	++	++
LB16	0.24 ± 0.02 ^a^	++	+	−	+	−	−	15.24 ± 0.32 ^d^	13.71 ± 0.2 ^a^	8.73 ± 0.33 ^d^	+	++	+
LB3	0.03 ± 0.02 ^a^	-	+	+	+	+	+	7.79 ± 0.24 ^b^	6.63 ± 32 ^b^	3.90 ± 0.29 ^b^	++	++	++
AS10	0.38 ± 0.04 ^a^	-	−	−	−	−	−	11.10 ± 0.33 ^c^	0.86 ± 0.3 ^a^	5.13 ± 0.30 ^c^	+	+	+
i40C	0.01 ± 0.006 ^a^	-	−	−	−	−	−	7.34 ± 0.33 ^b^	12.71 ± 0.33 ^c^	33.93 ± 0.33 ^g^	+	++	++
AS9	0.02 ± 0.01 ^a^	-	−	−	−	−	−	54.04 ± 0.33 ^f^	14.40 ± 0.33 ^c^	36.07 ± 0.33 ^h^	+	+	+
LB6	0.13 ± 0.0.07 ^a^	+	+	+	−	−	−	28.71 ± 0.13 ^e^	40.18 ± 0.33 ^d^	23.63 ± 0.33 ^f^	+	++	++

^a^ + positive with a smaller clear zone; ++ positive with a larger clear zone; − negative. ^b^ Each data point is expressed as the mean of at least three replicates. ^c–h^ Different superscript letters indicate significant differences (*p* < 0.05) among the values in the same column.

**Table 2:** Significance levels (F-values) of treatments and treatment interactions on measured variables as determined using LSD tests (*, **, ***, and ns indicate significant differences at *p* < 0.05, *p* < 0.01, *p* < 0.001, and non-significant differences, respectively).

Source of Variation	Heat Stress	Inoculation	Heat Stress × Inoculation
Shoot length	1986 ***	19 ***	2 *
Root length	370 ***	2 ns	3 **
Shoot dry weight	800 ***	777 ***	297 ***
Root dry weight	1681 ***	961 ***	625 ***
Chlorophyll SPAD	172 ***	8 ***	0.9 ns
Proline	139 ***	12 ***	15 ***
MDA content	23 ***	3 *	2 *
Abscisic acid	19 ***	1.8 ns	1.9 ns

## Data Availability

The original contributions presented in this study are included in this article/Supplementary Material. The sequence data analyzed in this study were deposited in the GenBank database under Accession Numbers MZ144276, MZ144406, MZ144407, MZ144408, MZ144255, MZ144252, MZ144277, MZ144253, MZ340553, MZ340638, MZ340514, MZ340515, MZ144251, MZ144249, MZ144254, MZ144250, MZ144256, MZ340516, MZ340517, MZ340279, MZ340274, MZ340278, MZ144275, MZ144280, and MZ144281. Further inquiries can be directed to the corresponding authors.

## Supplementary Materials

The following supporting information can be downloaded at: https://www.mdpi.com/article/10.3390/microorganisms13040766/s1. Table S1: Effect of thermotolerance endophytic strains isolated from Taiwan rice seeds on growth under seed germination bioassay (5-h heat shock at 45 °C after growing at 25 °C for 5 d and later 5 more d at 25 °C). BK means no inoculation; NG means no heat shock and no inoculation; Table S2: Effect of thermotolerance endophytic strains isolated from Taiwan rice seeds on growth under seed germination bioassay (5-h heat shock at 40 °C after growing at 25 °C for 5 d and later 5 more d at 25 °C). BK means no inoculation; NG means no heat shock and no inoculation; Table S3: 16S rRNA gene sequence-based identification of 25 cultured Tainan no. 11 rice seed endophytic bacterial strains that showed best results in Petri dish test.

## References

[1] Tripathy S., Meena S., Babu S., Das T., Dhar S. (2021). Phosphorus pools under integrated phosphorus management of upland direct-seeded rice (*Oryza sativa*) in North-Eastern Hill region of India. Indian J. Agron..

[2] Awasthi A., Pattnayak K.C., Tandon A., Sarkar A., Chakraborty M. (2023). Implications of climate change on surface temperature in North Indian states: Evidence from CMIP6 model ensembles. Front. Environ. Sci..

[3] Anderson R., Bayer P.E., Edwards D. (2020). Climate change and the need for agricultural adaptation. Curr. Opin. Plant Biol..

[4] Abbas S., Mayo Z.A. (2021). Impact of temperature and rainfall on rice production in Punjab, Pakistan. Environ. Dev. Sustain..

[5] Munakata Y., Spina R., Slezack-Deschaumes S., Genestier J., Hehn A., Laurain-Mattar D. (2022). Screening of endophytic bacteria of Leucojum aestivum ‘gravety giant’ as a potential source of alkaloids and as antagonist to some plant fungal pathogens. Microorganisms.

[6] Ganie S.A., Bhat J.A., Devoto A. (2022). The influence of endophytes on rice fitness under environmental stresses. Plant Mol. Biol..

[7] Compant S., Cambon M.C., Vacher C., Mitter B., Samad A., Sessitsch A. (2021). The plant endosphere world–bacterial life within plants. Environ. Microbiol..

[8] Haldar S., Sengupta S. (2023). An Overview of the Multifaceted Role of Plant Growth-Promoting Microorganisms and Endophytes in Sustainable Agriculture: Developments and Prospects. Microbial Symbionts and Plant Health: Trends and Applications for Changing Climate.

[9] Khan M.A., Asaf S., Khan A.L., Jan R., Kang S.-M., Kim K.-M., Lee I.-J. (2020). Thermotolerance effect of plant growth-promoting Bacillus cereus SA1 on soybean during heat stress. BMC Microbiol..

[10] Zhang Q., White J.F. (2021). Bioprospecting desert plants for endophytic and biostimulant microbes: A strategy for enhancing agricultural production in a Hotter, drier future. Biology.

[11] Liang Z., Lin X., Liao Y., Tang T. (2022). Characteristics and diversity of endophytic bacteria in Panax notoginseng under high temperature analysed using full-length 16S rRNA sequencing. Arch. Microbiol..

[12] Sharma P., Suman A., Aswini K., SaiPrasad J., Gond S. (2023). Endophytic bacterial taxonomic and functional diversity in the seeds of wheat genotypes from different agroecologies. J. Plant Interact..

[13] Devi R., Kaur T., Negi R., Kour D., Chaubey K.K., Yadav A.N. (2023). Indigenous plant growth-promoting rhizospheric and endophytic bacteria as liquid bioinoculants for growth of sweet pepper (*Capsicum annuum* L.). Biologia.

[14] Zhang M., Yang L., Hao R., Bai X., Wang Y., Yu X. (2020). Drought-tolerant plant growth-promoting rhizobacteria isolated from jujube (*Ziziphus jujuba*) and their potential to enhance drought tolerance. Plant Soil.

[15] Checinska Sielaff A., Urbaniak C., Mohan G.B.M., Stepanov V.G., Tran Q., Wood J.M., Minich J., McDonald D., Mayer T., Knight R. (2019). Characterization of the total and viable bacterial and fungal communities associated with the International Space Station surfaces. Microbiome.

[16] Rodríguez-Rodríguez R.M., Guimarães A.A., de Castro J.L., Siqueira J.O., Carneiro M.A.C., de Souza Moreira F.M. (2021). Rhizobia and endophytic bacteria isolated from rainforest fragments within an iron ore mining site of the Eastern Brazilian Amazon. Braz. J. Microbiol..

[17] Montes-Luz B., Conrado A.C., Ellingsen J.K., Monteiro R.A., de Souza E.M., Stacey G. (2023). Acetylene Reduction Assay: A Measure of Nitrogenase Activity in Plants and Bacteria. Curr. Protoc..

[18] Berza B., Sekar J., Vaiyapuri P., Pagano M.C., Assefa F. (2022). Evaluation of inorganic phosphate solubilizing efficiency and multiple plant growth promoting properties of endophytic bacteria isolated from root nodules *Erythrina brucei*. BMC Microbiol..

[19] Singh P., Kardile H.B., Rawal S., Kumar M., Dua V., Sharma J., Kumar S. (2022). Beneficial Effects of Bacterial Endophytes Isolated From Potato Roots And Tubers on Nutrient Solubilization. Potato J..

[20] Fiodor A., Ajijah N., Dziewit L., Pranaw K. (2023). Biopriming of seed with plant growth-promoting bacteria for improved germination and seedling growth. Front. Microbiol..

[21] Alemneh A.A., Cawthray G.R., Zhou Y., Ryder M.H., Denton M.D. (2021). Ability to produce indole acetic acid is associated with improved phosphate solubilising activity of rhizobacteria. Arch. Microbiol..

[22] Schwyn B., Neilands J. (1987). Universal chemical assay for the detection and determination of siderophores. Anal. Biochem..

[23] Chowdappa S., Jagannath S., Konappa N., Udayashankar A.C., Jogaiah S. (2020). Detection and characterization of antibacterial siderophores secreted by endophytic fungi from Cymbidium aloifolium. Biomolecules.

[24] Goyal K., Singh N., Jindal S., Kaur R., Goyal A., Awasthi R. (2022). Kjeldahl method. Adv. Tech. Anal. Chem..

[25] Hameed A., Chen Y.-P., Shen F.-T., Lin S.-Y., Huang H.-I., Lin Y.-W., Young C.-C. (2023). Evaluation of a subtropical maize-rice rotation system maintained under long-term fertilizer inputs for sustainable intensification of agriculture. Appl. Soil Ecol..

[26] Tiwari S., Lata C., Chauhan P.S., Nautiyal C.S.J.P.P. (2016). Biochemistry. Pseudomonas putida attunes morphophysiological, biochemical and molecular responses in *Cicer arietinum* L. during drought stress and recovery. Plant Physiol. Biochem..

[27] Lin S.-Y., Hameed A., Tsai C.-F., Young C.-C. (2023). Description of *Flavobacterium agricola* sp. nov., an auxin producing bacterium isolated from paddy field. Antonie Van Leeuwenhoek.

[28] Zhang H., Tu Y., Kang J., Song W., Zheng L. (2021). Blue light dosage affects photosynthesis, chlorophyll, and antioxidant properties of *Mesembryanthemum crystallinum*. Photosynthetica.

[29] Alexander A., Singh V.K., Mishra A. (2020). Halotolerant PGPR Stenotrophomonas maltophilia BJ01 induces salt tolerance by modulating physiology and biochemical activities of *Arachis hypogaea*. Front. Microbiol..

[30] Williams P., De Mallorca M.S. (1982). Abscisic acid and gibberellin-like substances in roots and root nodules of *Glycine max*. Plant Soil.

[31] Castro-Cegrí A., Sierra S., Hidalgo-Santiago L., Esteban-Muñoz A., Jamilena M., Garrido D., Palma F. (2023). Postharvest Treatment with Abscisic Acid Alleviates Chilling Injury in Zucchini Fruit by Regulating Phenolic Metabolism and Non-Enzymatic Antioxidant System. Antioxidants.

[32] Cummins W., Sondheimer E.J.P. (1973). Activity of the asymmetric isomers of abscisic acid in a rapid bioassay. Planta.

[33] Yang J., Zhou Q., Shen K., Song N., Ni L. (2018). Controlling nanodomain morphology of epoxy thermosets templated by poly (caprolactone)-block-poly (dimethylsiloxane)-block-poly (caprolactone) ABA triblock copolymer. RSC Adv..

[34] Arachchige S.M., Razzaq A., Dai H.-Y., Wang J. (2024). Confronting Heat Stress in Crops Amid Global Warming: Impacts, Defense Mechanisms, and Strategies for Enhancing Thermotolerance. Crop Breed. Genet. Genom..

[35] Mishra S., Spaccarotella K., Gido J., Samanta I., Chowdhary G. (2023). Effects of heat stress on plant-nutrient relations: An update on nutrient uptake, transport, and assimilation. Int. J. Mol. Sci..

[36] Gupta A., Mishra R., Rai S., Bano A., Pathak N., Fujita M., Kumar M., Hasanuzzaman M. (2022). Mechanistic insights of plant growth promoting bacteria mediated drought and salt stress tolerance in plants for sustainable agriculture. Int. J. Mol. Sci..

[37] Islam M.N., Masud A.A.C., Alam M.M., Islam M.N., Rahman M.L., Hasanuzzaman M. (2022). Osmolyte-induced water deficit stress mitigation during panicle initiation stage in transplanted rice (*Oryza sativa* L.). Horizon.

[38] Zulfiqar F., Ashraf M. (2023). Proline alleviates abiotic stress induced oxidative stress in plants. J. Plant Growth Regul..

[39] Shaffique S., Khan M.A., Wani S.H., Pande A., Imran M., Kang S.-M., Rahim W., Khan S.A., Bhatta D., Kwon E.-H. (2022). A review on the role of endophytes and plant growth promoting rhizobacteria in mitigating heat stress in plants. Microorganisms.

[40] Anas M., Khalid A., Saleem M.H., Ali Khan K., Ahmed Khattak W., Fahad S. (2025). Symbiotic Synergy: Unveiling Plant-Microbe Interactions in Stress Adaptation. J. Crop Health.

[41] Lastochkina O., Garshina D., Allagulova C., Fedorova K., Koryakov I., Vladimirova A. (2020). Application of endophytic Bacillus subtilis and salicylic acid to improve wheat growth and tolerance under combined drought and Fusarium root rot stresses. Agronomy.

[42] Sun X., Xu Z., Xie J., Hesselberg-Thomsen V., Tan T., Zheng D., Strube M.L., Dragoš A., Shen Q., Zhang R. (2022). Bacillus velezensis stimulates resident rhizosphere Pseudomonas stutzeri for plant health through metabolic interactions. ISME J..

[43] Carreiras J., Cruz-Silva A., Fonseca B., Carvalho R.C., Cunha J.P., Proença Pereira J., Paiva-Silva C., Santos S.A., Janeiro Sequeira R., Mateos-Naranjo E. (2023). Improving Grapevine Heat Stress Resilience with Marine Plant Growth-Promoting Rhizobacteria Consortia. Microorganisms.

[44] Notununu I., Moleleki L., Roopnarain A., Adeleke R. (2022). Effects of plant growth-promoting rhizobacteria on the molecular responses of maize under drought and heat stresses: A review. Pedosphere.

[45] Sharma M.M.M., Sharma P., Kapoor D., Beniwal P., Mehta S. (2021). Phytomicrobiome community: An agrarian perspective towards resilient agriculture. Plant Performance Under Environmental Stress: Hormones, Biostimulants and Sustainable Plant Growth Management.

[46] Muhammad Aslam M., Waseem M., Jakada B.H., Okal E.J., Lei Z., Saqib H.S.A., Yuan W., Xu W., Zhang Q. (2022). Mechanisms of abscisic acid-mediated drought stress responses in plants. Int. J. Mol. Sci..

[47] Sharma K., Jalota K., Agarwal C., Pal P., Jindal S. (2024). Biochemical Responses of Plants to Individual and Combined Abiotic Stresses. Plant Secondary Metabolites and Abiotic Stress.

[48] Ahmad H.M., Fiaz S., Hafeez S., Zahra S., Shah A.N., Gul B., Aziz O., Fakhar A., Rafique M., Chen Y. (2022). Plant growth-promoting rhizobacteria eliminate the effect of drought stress in plants: A review. Front. Plant Sci..

[49] Xiong R., Chen Y. (2025). Molecular mechanisms and nutrient regulation of crop root responses to drought stress: Interactions with rhizosphere microorganisms. Sustainable Agriculture Under Drought Stress.

[50] Khan M.S., Zulfiqar I. (2023). Microbial mitigation of drought stress in plants: Adaptations to climate change. Abiotic Stress in Plants—Adaptations to Climate Change.

[51] Al-Turki A., Murali M., Omar A.F., Rehan M., Sayyed R. (2023). Recent advances in PGPR-mediated resilience toward interactive effects of drought and salt stress in plants. Front. Microbiol..

[52] Ha-Tran D.M., Nguyen T.T.M., Hung S.-H., Huang E., Huang C.-C. (2021). Roles of plant growth-promoting rhizobacteria (PGPR) in stimulating salinity stress defense in plants: A review. Int. J. Mol. Sci..

