# Supplementary material for: Unveiling the Thermotolerance and Growth-Promoting Attributes of Endophytic Bacteria Derived from Oryza sativa: Implications for Sustainable Agriculture
Source: Microorganisms. 2025 Mar 27;13(4):766. doi: 10.3390/microorganisms13040766 (PMC12029165; doi:10.3390/microorganisms13040766)
Supplement: Supplementary file 1 [file microorganisms-13-00766-s001.zip › microorganisms-3540892-supplementary.pdf]

Supplementary Materials

Table S1. Effect of thermotolerance endophytic strains isolated from Taiwan rice seeds on growth under seed germination bioassay (5-h heat shock at 45℃ after growing at 25℃ for 5 d and later 5 more d at 25 ℃). BK means no inoculation; NG means no heat shock and no inoculation.

Isolates	Length (cm)		Fresh weight (mg plant^{-1})	
	Hypocotyl	Radicle	Hypocotyl	Radicle
BK	2.68±0.12	5.79±0.28	12.49±0.86	4.99±0.48
DF1	3.56±0.12	8.26±0.28	17.25±0.84	11.28±0.75*
DF2	3.93±0.11	9.37±0.29	22.51±0.62*	12.92±0.46***
DF3	3.93±0.15	9.42±0.33	21.05±1.22**	11.90±1.02**
DF4	3.15±0.11	7.54±0.39***	21.60±0.66**	14.15±0.99**
DF5	3.87±0.12	9.30±0.36***	19.10±0.80*	22.25±3.08***
DF6	3.64±0.10	9.57±0.30***	22.93±2.34	12.01±1.49**
DF7	3.57±0.12	9.92±0.30***	15.66±0.96	9.29±1.08
DF8	3.83±0.11	11.38±0.42***	12.67±0.87	5.21±0.53
DF9	4.07±0.15	9.94±0.36***	23.16±0.87***	13.18±0.95***
DF10	3.39±0.13	8.94±0.25*	19.27±0.84*	12.36±1.00**
DF11	3.69±0.10	10.98±0.41***	22.68±0.93**	18.78±3.18***
DF12	3.85±0.17	9.38±0.26***	20.90±0.99**	13.30±1.40***
DF13	2.92±0.11	7.21±0.29***	21.17±4.56**	11.75±2.30*
DF14	2.83±0.12	7.99±0.37***	19.80±0.76*	9.25±0.74
DF15	3.03±0.12	8.10±0.47***	25.83±5.16***	7.88±0.50
DF16	3.38±0.07***	8.79±0.41***	18.74±0.49*	7.64±0.86
DF17	3.86±0.15***	9.71±0.49***	23.52±1.23***	10.52±0.66*
DF18	4.25±0.11***	12.26±0.29***	37.75±2.40***	49.40±3.62***
DF19	4.29±0.10***	11.17±0.22***	26.76±2.65	31.70±3.15***
DF20	3.16±0.08***	8.66±0.36***	19.05±4.39*	3.81±1.08
DF21	4.29±0.10***	11.03±0.26***	20.19±0.64**	4.42±0.70
DF22	3.39±0.17*	9.82±0.28***	15.04±0.76*	1.81±0.28
DF23	3.78±0.16***	10.94±0.35***	17.05±2.95*	1.96±0.23
E45C	5.7±0.14***	9.91±0.30***	35.74±0.85***	26.29±1.17***
AS1	5.45±0.16***	8.50±0.40	22.74±0.87	18.56±0.96***
F45C	6.32±0.15***	10.88±0.28***	36.90±1.64***	37.26±2.59
AS2	4.92±0.15*	8.74±0.42*	26.06±1.06**	17.37±1.22***
a45C	6.83±0.17***	11.89±0.40***	36.02±0.94***	39.81±2.57***
AS3	5.53±0.31***	9.45±0.37**	23.65±0.77	8.80±0.49
b45C	6.04±0.13***	13.86±0.48***	32.79±1.12***	20.92±1.06***
AS4	5.36±0.18***	8.36±0.45***	21.10±0.88***	18.16±1.09***
c5C	6.29±0.17***	11.47±0.34***	30.87±0.85***	17.149±1.28***
AS5	5.84±0.41***	10.97±0.38***	32.25±1.08***	18.50±1.09***
d45C	6.28±0.12***	9.50±0.40***	33.74±0.96***	26.79±1.94***
AS6	5.57±0.17***	10.13±0.48***	32.27±0.77***	17.95±0.90***
E45C	5.93±0.13***	9.95±0.26***	36.74±0.91***	32.32±2.48***
AS7	5.58±0.18***	11.85±0.35***	28.13±1.26***	20.70±1.15***
AS8	5.33±0.15***	10.23±0.45***	34.06±1.09***	32.60±1.71**
AS9	5.83±0.16***	10.67±0.44	36.66±1.25***	36.13±1.73***
AS10	5.52±0.15***	10.45±0.45***	34.23±1.70***	27.49±1.56***
AS11	5.69±0.16***	9.18±0.44***	33.77±1.13***	37.25±2.19***
LB1	6.06±0.26***	10.91±0.41***	27.82±0.94***	22.88±1.70***

LB2	4.72±0.09	9.84±0.50***	24.35±1.19***	16.04±1.18***
LB3	5.97±0.11***	12.69±0.29***	36.39±6.47***	22.07±1.77***
LB4	4.18±0.13	11.82±0.76***	27.75±1.30***	20.29±1.44***
LB5	3.63±0.12**	10.34±0.40***	32.03±1.60***	22.98±1.90***
LB6	5.96±0.21***	13.86±0.36***	35.67±2.54***	29.99±1.39***
LB7	3.36±0.06***	11.66±0.32***	29.89±1.78***	20.35±1.31***
LB8	5.17±0.13*	12.70±0.30**	28.02±1.04***	21.67±1.50***
LB9	3.87±0.08***	11.44±0.31***	32.47±1.32***	27.36±1.35***
LB10	6.45±0.10***	14.93±0.34***	36.51±1.31***	29.45±1.52***
a45C	5.13±0.14**	8.83±0.35*	30.88±1.07***	19.25±0.88***
b45C	5.77±0.13***	9.85±0.37***	33.72±1.36***	29.47±2.78***
C45C	5.71±0.14***	11.72±0.39***	34.76±1.33***	19.92±0.86***
D45C	5.96±0.12***	10.11±0.46***	32.90±1.04***	19.96±1.19***
NG	5.65±0.16***	10.39±0.31***	25.06±0.84***	18.07±0.72***

All the values are expressed as mean ± standard error calculated from 50 germinated seeds. Statistically significant difference of each mean from BK is indicated as * P<0.05, ** P<0.01 or *** P<0.001(P, Fisher's LSD).

Table S2. Effect of thermotolerance endophytic strains isolated from Taiwan rice seeds on growth under seed germination bioassay (5-h heat shock at 40℃ after growing at 25℃ for 5 d and later 5 more d at 25 ℃). BK means no inoculation; NG means no heat shock and no inoculation.

Isolates	Length (cm)		Fresh weight (mg plant^{-1})	
	Hypocotyl	Radicle	Hypocotyl	Radicle
BK	3.36±0.14	7.05±0.48	18.90±1.27	14.17±1.29
DF24	3.98±0.11***	10.86±0.32***	17.16±0.81	20.69±4.42*
DF25	3.78±0.10*	10.88±0.32***	19.55±0.58	12.66±1.91
DF26	3.85±0.06*	10.91±0.40**	22.04±0.48*	7.57±0.65
DF27	4.42±0.12**	11.38±0.30***	26.68±4.21***	10.66±0.44
DF28	3.73±0.12***	11.14±0.43**	19.66±0.74	6.88±0.30
DF29	3.96±0.09***	10.68±0.32***	23.53±0.50*	15.99±3.20
DF30	3.86±0.15**	9.19±0.41***	26.12±0.85***	14.88±0.66
DF31	4.13±0.11***	12.42±0.27	28.86±0.81***	26.80±5.35***
DF32	3.64±0.14	8.44±0.42	28.24±4.40***	10.22±0.34
DF33	4.42±0.08***	11.86±0.21	29.31±0.70***	25.28±5.22***
DF34	3.75±0.14*	11.07±0.32	27.25±0.79	15.76±0.70
DF35	3.99±0.17***	10.56±0.42	25.84±2.65	17.81±2.02
DF36	5.21±0.15***	13.16±0.28	34.30±0.89	22.27±0.95*
DF37	4.51±0.10**	12.91±0.19***	31.15±0.71	20.49±2.35*
DF38	3.67±0.11***	5.64±0.20	20.85±0.95	17.02±0.99
DF39	4.21±0.14***	5.14±0.25	20.77±0.99	15.49±1.41
DF40	4.08±0.07***	7.94±0.39	24.56±0.88**	22.49±1.18**
DF41	4.27±0.08***	6.22±0.33	22.65±0.91	22.20±1.55
DF42	4.41±0.12***	6.71±0.34	21.55±0.61	19.18±1.30
DF43	4.98±0.16***	6.55±0.31	27.23±1.13***	19.32±0.80
DF44	4.64±0.14***	8.82±0.50**	17.92±1.84***	17.97±1.63
DF45	4.59±0.09***	7.92±0.40	26.71±0.81	26.40±2.60**
DF46	4.71±0.08***	8.49±0.42	27.69±1.80***	21.54±1.29**
DF47	4.81±0.12***	9.05±0.50***	30.09±0.99***	26.85±1.59***
DF48	4.67±0.10***	9.58±0.38***	25.98±0.80	21.09±1.61*
AS18	5.00±0.15	10.49±0.37***	34.85±1.39	18.07±1.82
LB11	6.71±0.08***	12.95±0.28***	47.89±1.76	43.79±1.88**
AS19	6.35±0.20***	13.98±0.27***	31.91±0.92	19.29±0.80
LB12	6.70±0.10***	13.19±0.34***	44.23±1.33***	22.56±0.97
AS20	5.78±0.12	14.54±0.39***	33.85±1.05	18.69±1.51
LB13	6.93±0.09***	12.54±0.35***	37.85±0.97***	26.33±1.31***
AS21	6.37±0.16***	13.43±0.34***	46.40±1.08	24.61±0.96**
LB14	6.56±0.11***	12.55±0.29***	43.73±1.01***	32.70±1.17
LB15	6.64±0.07***	14.40±0.29***	52.99±1.18	39.09±1.54
AS22	5.92±0.16***	13.78±0.31***	41.11±1.08**	23.63±0.92**
LB16	6.70±0.09**	13.95±0.31***	47.39±1.15	35.65±1.51
AS23	6.75±0.13	14.32±0.34***	51.44±0.98	33.96±1.40

LB17	4.72±0.19	9.15±0.43	48.51±1.32	27.11±0.67
AS24	6.42±0.15	14.23±0.26***	44.61±0.87	30.52±0.91
LB18	5.46±0.14	9.83±0.43**	41.77±1.08***	22.83±0.61***
AS25	6.59±0.20***	13.32±0.51	50.65±1.19***	33.35±0.95
LB19	5.69±0.12	11.23±0.27	48.11±1.87***	40.77±2.38
AS26	5.65±0.19	13.24±0.48***	43.91±1.51***	33.82±1.20***
LB20	5.37±0.12***	9.45±0.46***	41.56±0.78***	23.15±0.84***
AS27	6.62±0.18***	13.84±0.30***	44.83±1.36***	24.45±1.36***
LB21	6.50±0.14***	11.83±0.40***	41.58±1.38***	31.24±1.66***
AS28	5.46±0.18***	11.45±0.55***	31.06±1.04***	7.64±0.62***
LB22	5.83±0.15	7.83±0.39	33.81±1.35***	17.81±0.78
LB23	5.34±0.15	9.27±0.51**	34.52±1.14***	11.71±0.51
B40c	7.12±0.21	12.79±0.33***	33.88±0.71	19.90±1.55
LB24	6.89±0.23	11.62±0.36***	38.01±1.46***	29.39±2.63
c40C	7.17±0.14	14.59±0.37***	35.00±0.73***	16.41±0.58
LB25	5.68±0.15	8.34±0.37***	29.53±1.04***	7.53±0.81
g40C	8.06±0.18	13.51±0.35***	47.94±1.27***	25.38±2.29**
LB26	6.14±0.13***	10.14±0.38**	33.53±1.17***	18.74±1.33
40C	7.58±0.18***	13.80±0.35***	40.81±1.00***	24.67±1.09**
LB27	5.33±0.09***	10.41±0.40**	28.90±1.08	5.43±0.27***
i40C	6.91±0.14***	14.78±0.35	36.68±1.01	24.59±2.22
LB28	6.00±0.18	8.55±0.31***	30.10±1.10	17.90±1.03
j40C	6.76±0.12***	14.64±0.30***	34.30±1.35	17.76±1.13
LB29	5.60±0.34	8.63±0.38***	25.53±1.54	4.72±0.70
d40C	6.01±0.14*	13.29±0.44***	35.03±1.16	19.66±1.76
AS12	5.60±0.21	9.49±0.33	22.89±1.13	5.04±0.28
f40C	5.57±0.12*	12.17±0.60***	31.54±0.91	16.82±1.09
AS13	6.18±0.16***	12.21±0.35***	33.91±0.89	15.05±0.60**
g40C	4.84±0.20*	10.85±0.43***	27.01±1.27	15.87±1.18*
AS14	6.03±0.18**	10.75±0.37**	22.43±0.94***	6.87±1.38***
j40C	6.26±0.12***	13.14±0.38***	36.08±0.88***	15.18±1.28*
AS15	5.74±0.18	10.34±0.36**	28.90±0.88*	8.43±0.73***
k40C	5.54±0.14	12.55±0.33***	33.39±1.32**	19.67±1.31
AS16	6.93±0.17***	13.92±0.42***	45.28±1.80***	34.20±0.97***
AS17	6.49±0.14***	12.53±0.27***	35.52±0.94	17.47±0.68
NG	5.65±0.16***	10.39±0.31***	25.06±0.84	18.07±0.72

All the values are expressed as mean ± standard errror from 50 germinated seeds. Statistically significant difference of each mean from BK is indicated as * P<0.05, ** P<0.01 or *** P<0.001 (Fisher’s LSD).

Table S3. 16S rRNA gene sequence-based identification of 25 cultured Tainan no. 11 rice seed endophytic bacterial strains that showed best results in Petri dish test

Strain name	Closest match	% Similarity (Δbp)	Variation ratio
AS25	*Bacillus tequilensis*	99.93	1420/1421
AS23	*Bacillus tequilensis*	99.86	1427/1429
AS10*	*Bacillus haynesii*	99.71	1398/1402
AS9*	*Bacillus paralicheniformis*	99.64	1368/1373
AS5	*Bacillus tequilensis*	99.93	1420/1421
LB24	*Bacillus tequilensis*	99.86	1423/1425
LB19	*Bacillus tequilensis*	99.43	1407/1415
LB16*	*Bacillus pumilus*	99.72	1420/1424
LB15	*Bacillus safensis*	99.86	1423/1425
LB11	*Bacillus tequilensis*	99.86	1432/1434
LB10	*Bacillus tequilensis*	99.86	1418/1420
LB6*	*Bacillus coagulans*	98.73	1402/1420
LB3*	*Bacillus tequilensis*	99.72	1425/1429
DF48	*Bacillus velezensis*	99.78	1386/1389
DF47	*Bacillus safensis*	99.93	1421/1422
DF36*	*Bacillus velezensis*	99.93	1386/1387
DF31	*Bacillus velezensis*	99.92	1209/1210
DF19	*Bacillus safensis*	100	1420/1420
DF18*	*Bacillus velezensis*	99.93	1386/1387
DF9	*Bacillus velezensis*	99.71	1386/1390
DF3	*Bacillus velezensis*	99.93	1384/1385
i40C*	*Bacillus paranthracis*	99.93	1420/1421
G40c	*Bacillus safensis*	99.86	1426/1428
f45C	*Bacillus safensis*	99.86	1425/1427
a45C	*Bacillus safensis*	99.79	1427/1430

[a] Strains with asterisk * were selected for PGPT test
